# Health status of and health-care provision to asylum seekers in Germany: protocol for a systematic review and evidence mapping of empirical studies

**DOI:** 10.1186/2046-4053-3-139

**Published:** 2014-11-29

**Authors:** Christine Schneider, Amir Mohsenpour, Stefanie Joos, Kayvan Bozorgmehr

**Affiliations:** Department of General Practice and Health Services Research, University Heidelberg, Voßstraße 2, Gebäude 37, D-69115 Heidelberg, Germany

**Keywords:** Asylum seekers, Refugees, Germany, Health status, Health-care provision, Access, Health services, Systematic review, Evidence mapping

## Abstract

**Background:**

There are more than 100,000 asylum seekers registered in Germany, who are granted limited access to health services. This study aims to provide a systematic overview of the empirical literature on the health status of and health-care provision to asylum seekers in Germany in order to consolidate knowledge, avoid scientific redundance, and identify research gaps.

**Methods/design:**

A systematic review and evidence mapping of empirical literature on the health status of and health-care provision to asylum seekers in Germany will be performed. We will apply a three-tiered search strategy: 1. search in databases (PubMed/MEDLINE, Web of Science, IBSS, Sociological Abstracts, Worldwide Political Science Abstracts, CINAHL, Sowiport, Social Sciences Citation Index, ASSIA, MedPilot, DNB), dissertation and theses databases, and the internet (Google); 2. screening references of included studies; 3. contacting authors and civil society organizations for grey literature. Included will be studies which report quantitative and/or qualitative data or review articles on asylum seekers in Germany, published in German or English language. Outcome measures will include physical, mental, or social well-being, and all aspects of health-care provision (access, availability, affordability, and quality). Search results will be screened for eligibility by screening titles, abstracts and full texts. Data extraction comprises information on study characteristics, research aims, and domains of health or health-care services analyzed. The quality of studies will be appraised and documented by appropriate assessment tools. A descriptive evidence map will be drawn by categorizing all included articles by research design and the health conditions and/or domains of health-care provision analyzed. The body of evidence will be evaluated, and a narrative evidence synthesis will be performed by means of a multi-level approach, whereby quantitative and qualitative evidence are analyzed as separate streams and the product of each stream is configured in a final summary.

**Discussion:**

This systematic review will provide an evidence map and synthesis of available research findings on the health status of and health-care provision to asylum seekers in Germany. In anticipation of identifying areas which are amenable to health-care interventions, deserve immediate action, or further exploration, this review will be of major importance for policy-makers, health-care providers, as well as researchers.

**Systematic review registration:**

PROSPERO 2014: CRD42014013043

**Electronic supplementary material:**

The online version of this article (doi:10.1186/2046-4053-3-139) contains supplementary material, which is available to authorized users.

## Background

In 2013, the number of asylum seekers and internally displaced people (IDP) worldwide exceeded 50 million people—for the first time after World War II. The latest annual Global Trends Report (June 2014) of the United Nations Refugee Agency (UNHCR) shows that 51.2 million people were forcibly displaced at the end of 2013 as a result of persecution, conflict, generalized violence, or human rights violations[1].

The huge number of 51.2 million displaced people includes 33.3 million IDP and 16.7 million refugees, with more than half (53%) of all refugees worldwide coming from just three countries: Afghanistan, Syria, and Somalia. Of all the refugees, 86% are being hosted by developing countries with Pakistan, Iran, and Lebanon hosting the largest numbers. In addition to refugees and IDP, 1.2 million individuals are estimated to be seeking international protection and whose claim for refugee status has not yet been determined (asylum seekers).

The majority (51%) of asylum seekers were registered in industrialized countries, mostly due to political developments in countries of origin, changes in asylum policies, and practices in receiving countries and the fact that some countries are perceived as being more likely to grant refugee status than others[2].

For the first time since 1999, Germany was the world’s largest recipient of new individual applications, with a total of 127,023 asylum claims—followed by USA and South Africa[1]. Hence, the German health system faces new challenges by guaranteeing adequate health-care provision to the rising number of asylum seekers.

Access to health care for asylum seekers in Germany is governed via the national Law on Services for Asylum Seekers (AsylBLG sections 4 and 6). The law is broad in conception, but in practice, it limits access to health services and emergency care services for this population and links it with lengthy bureaucratical procedures[3].

This problem is long known and has been addressed by civil society organizations and health professionals through initiatives on different societal levels, ranging from political campaigns to the provision of solidarity health-care services. Furthermore, the living conditions of asylum seekers, the post-migration factors affecting their health and wellbeing, as well as the difficulties they face in accessing health services have repeatedly been subject to public debate and scientific inquiry by scientists of different disciplines.

These inquiries are particularly important because the routine health information system in Germany does not capture information related to the health status of and health-care provision to asylum seekers. This means that basically no information on this population group can be derived from routine health monitoring sources. Data protection issues (e.g., lack of information on residence status in claims data) further hamper the possibility to assess, e.g., health problems or access to and utilization of health services on a routine basis.

Despite the longstanding public health relevance of this topic, there is yet no systematic overview of the research landscape on the health status of and health-care provision to asylum seekers in Germany. The lack of overview of this field makes it difficult to judge upon the range, scope, and quality of research conducted. It also limits the possibility to develop the prospects of a future research agenda.

A pertinent example can be found in the literature on mental health of asylum seekers. Of the international systematic reviews on the prevalence of mental health disorders among asylum seekers known to the authors[4, 5], none list any studies conducted in Germany. It remains unclear whether this is due to a general lack of studies from Germany or due to the specific (quality-related) inclusion criteria of respective reviews. Further examples can be found in analytical studies conducted in other countries, which highlight the importance of potentially modifiable post-migration determinants affecting the health status of asylum seekers in their host countries. A longitudinal study conducted in the Netherlands has shown that frequent re-locations between asylum seeker centers increase the risk for mental distress among asylum-seeking children[6]. A meta-analysis of primary studies reporting psychopathologies among asylum seekers has found that worse mental health is (among other factors) associated with institutional accommodation and restricted economic opportunities in the host country[7]. Others have more broadly assessed the relation between need and utilization of health services among asylum seekers in the United Kingdoms[8]. However, it remains unclear if similar (or other) questions have been addressed in Germany at all, and if yes, to which extent.

Given the crucial role of research in illuminating health disparities, an overview of the scientific literature in the German context is highly needed to consolidate knowledge, avoid scientific redundance, and identify research gaps with respect to this marginalized population.

The objectives of this study are:

1. To systematically review, examine, and map the range, scope, and quality of research on the health status of and health-care provision to asylum seekers in Germany.
2. To synthesize knowledge from empirical studies on the health status of and health-care provision to asylum seekers Germany
3. To identify evidence gaps and areas for future research.

### Review questions

The following review questions will be addressed:

1. What is the range, scope, and quality of research on asylum seekers’ health status and health-care situation in Germany?
2. What is known from these studies about the health status of and health-care provision to asylum seekers in Germany?
3. What are the evidence gaps with respect to the health status of and health-care provision to asylum seekers in Germany?

## Methods/design

### Study design

This systematic review will apply both aggregative and configurative approaches[9]. Aggregative reviews are seeking evidence to inform decisions and make statements by collecting empirical data. The interest of configurative reviews is more in examining the complexity and range of different concepts than “in seeking a single correct answer”[9]. To examine the variation in and complexity of different research approaches (objectives 1 and 3), we will mainly use configurative methods. The information, attained from included studies, will serve to consolidate existing concepts and develop new insights about the health situation of asylum seekers. The review contains aggregative elements for analyzing collected data in a realist synthesis, which answers objective 2. An evidence map will be created with the aim of describing the research field and to structure and interpret the following synthesis.

The development of the protocol was informed and guided by the “EPPI-Centre guidelines”[10], the “Cochrane Guidelines for Systematic Reviews of Health Promotion and Public Health Interventions”[11], the “CRD’s Guidance for Undertaking Reviews in Health Care”[12], and “Systematic Reviews in the Social Sciences. A Practical Guide”[13].

The report of our systematic review will adhere to “PRISMA-Equity 2012 Extension: Reporting Guidelines for Systematic Reviews with a Focus on Health Equity”[14] as far as applicable to give special consideration to the aspect of equity.

### Search method for identification of studies

In the first step we will search in databases for relevant articles fulfilling pre-defined inclusion criteria. Following that, we will perform a screening of the citing and cited references of all included articles.

#### Strategy 1: search databases

We developed the search strategy according to “Cochrane Handbook for Systematic Reviews of Interventions”[15]. The following electronic databases will be searched for studies and reviews:

- Bibliographic databases
  - ○ PubMed/MEDLINE
  - ○ ISI Web of Science
  - ○ International Bibliography of Social Sciences
  - ○ Sociological Abstracts
  - ○ Social Sciences Citation Index
  - ○ Worldwide Political Science Abstracts
  - ○ CINAHL
  - ○ Sowiport
  - ○ ASSIA
  - ○ MedPilot
  - ○ Deutsche Nationalbibliothek
  - ○ Cochrane Library (without limitation to specific databases)
- Dissertation and theses databases

The search terms (refugee* OR asylum*) AND (health* OR access OR utilization OR use) AND german* are searched for in the articles’ title, abstract, and key words. For databases providing publications in German language, we will use the search terms (Asyl* OR Flüchtling*) AND Gesundheit*.

Further searches will be conducted by using web search engines (Google) to include grey literature articles, which give essential information but are not published in journals. To limit the number of hits to the relevant ones, we will search for (Asyl* ODER Flüchtling*) UND (Gesundheit ODER Gesundheitsversorgung) UND (Studie ODER qual* ODER quant*) and (refugee* OR asylum*) AND (health* OR access OR utilization OR use) AND german*. The Google search will be conducted in three steps: first, screening all hits; second, we will search for .pdf files only, followed by a search for .doc files only.

Additional searches will be performed on websites of NGOs to increase the sensitivity of our search strategy.

An overview of the final search term combinations which will be applied for searching each database is available in Additional file1*.*

#### Strategy 2: searching in the selected articles

We will view the reference lists of all included publications. Moreover, we will use backward and forward citations for Web of Knowledge for all publications included at the end of the screening process.

#### Strategy 3: contacting experts, individuals, and non-governmental organizations

We will contact experts, authors of identified studies, and non-governmental organizations to identify grey literature.

### Selection of studies-eligibility criteria

Broadly spoken, all empirical studies that use a sample of asylum seekers will be included regardless of the research question as long as they report a health outcome or an outcome measure related to health-care provision.

#### Inclusion criteria

More specifically, we will include studies which fulfill the following criteria:

- Type of population: refugees and asylum seekers (studies on “migrants” will be included only if a clear distinction is made between “refugees and asylum seekers” and other forms of migration in analysis and reporting of data).
- Types of studies: we will include all published empirical materials, including qualitative studies (in-depth interviews, semi-structured interviews, focus groups, ethnographies, participatory action research) and quantitative studies (cohort and case-control studies, cross-sectional studies, descriptive surveys, studies using routine/secondary data as long as health outcomes or health-care provision aspects are addressed), as well as mixed-methods studies. Unpublished material will only feed into the review if the review team is granted full access to the reports. Since many studies might not be analytical in nature, we explicitly consider descriptive surveys for inclusion provided that sufficient information on data collection and analysis is provided.
- Types of articles: original articles and review articles, including systematic and narrative reviews (of qualitative and/or quantitative research). Authors of relevant conference abstracts will be contacted for full research reports. If available, they will be included in the review.
- Type of outcome measure:
  - ○ Health: including all criteria of WHO definition [16]: physical, mental, and social well-being
  - ○ All aspects of health-care provision (i.e., accessibility, availability, affordability, and quality of health care)
- Geographical area: studies conducted in Germany or studies giving account of asylum seekers’ experiences in Germany (even if they do not reside anymore in Germany during the conduct of the study)
- Date of publication: initial search without limitation, mapping, and synthesis will be performed by distinguishing between studies pre-1993 and post-1993 due to major changes in legislation in 1993.

#### Exclusion criteria

- Type of population: migrants without clear reference to asylum seeker status/refugee status
- Types of articles: commentaries, discussion papers, journalistic interviews, policy reports, books, conference proceedings, abstracts
- Type of outcome measure: studies assessing social situation without clear link to health understood as physical, mental, and social well-being
- Geographical area: studies conducted on asylum seekers outside Germany and/or where the primary focus is not their situation while they resided in Germany
- Articles not published in German or English

### Screening process

The screening process will be conducted in two steps:

1. Screening titles and abstracts
2. Screening full texts

#### Title and abstract screening

Two reviewers will independently screen 10% of the articles by title and abstract resulting from the search process (after exclusion of duplicates) and assess them for inclusion using the *a priori* defined criteria. If necessary, inclusion and exclusion criteria will be re-defined based on this initial screening before screening all articles. Subsequently, all article titles and abstract will be screened in duplicate using the (potentially re-defined) inclusion/exclusion criteria. Eligibility for inclusion from both the initial and subsequent screening process will be recorded in an Excel File/EndNote database. Discrepancies in judgements on eligibility will be solved by discussion in the review team. If discrepancies in judgement on eligibility exist in absence of clear exclusion criteria, we will obtain all publications as full text that are deemed relevant by at least one reviewer.

#### Full text screening

We will obtain the full text of all the references included after title and abstract screening. All files will be incorporated to a bibliographic database (EndNote). Two reviewers will independently read the full text of the previously selected articles and assess eligibility for inclusion. Disagreements will be discussed in the review team and only references judged as eligible by all will be included.

#### Screening of citations

Finally, for the publications included after the screening process (i.e., for all publications meeting the inclusion criteria), we will review the references which are cited by and cite our “relevant articles”. Furthermore we will screen all articles received by experts, authors of identified studies, and non-governmental organizations. They will also be screened by using the stages described above (title, abstract, and full text) with the same inclusion and exclusion criteria.

#### Sensitivity and specificity of search strategy and selection process

We will assess the specificity (false-positives: excluded articles divided by all identified hits from the search results) and sensitivity (false-negatives: eligible articles not identified by our search strategies) using a test set of articles at hand of the authors prior to conducting the systematic review (Additional file2).

### Data extraction and critical appraisal

Data extraction and critical appraisal will be conducted simultaneously and be piloted against a random sample of the included articles by two reviewers.

#### Data extraction

We have designed preliminary data extraction forms based on the STROBE and QOREC checklists for quantitative and qualitative studies, respectively, adapted to the specific characteristics of this review (Additional file3 and Additional file4). The data extraction forms include the following items:

- Generic bibliographic information (author, year published)
- Study characteristics (year of study/study period/research method)
- Study objectives/research questions
- Population and context characteristics
- Health condition (physical and mental health/social well-being) and/or health-care provision domain analyzed or explored
- For quantitative studies: exposures and co-variables on individual and/or contextual level, as well as measures of frequency/association for the analyzed outcomes
- For qualitative studies: major themes/minor themes as reported
- Results of the critical appraisal

Further details on the type of data we seek to extract from quantitative/qualitative studies are provided in Additional file3 and Additional file4. The preliminary data extraction form for reviews can be found in Additional file5. Modifications in the data extraction forms are expected after the pilot study. Given the broad research questions, an iterative approach in developing and refining the data extraction form will be more adequate than a pre-defined fixed approach. All the reviewers will participate in the data extraction. Of the selected articles, 50% will be extracted by AM, the other 50% will be extracted by CS. All articles will be checked vice versa and will be checked by random sampling by SJ and KB. Disagreement will be resolved by discussion until consensus is reached. All extracted data will be recorded in a transparent and systematic way which will be detailed in the review report.

#### Critical appraisal

Different types of included studies need to be critically appraised with appropriate appraisal tools for the study design. Critical appraisal of selected quantitative studies will be conducted by means of the Quality Assessment Tool for Quantitative Studies of the Effective Public Health Practice Project (EPHPP) (see Additional file6). Mixed-methods studies will be judged by the *McGill Mixed-Methods Appraisal Tool for Mixed-Methods Studies*[8] (see Additional file7). The quality of reviews will be appraised using the AMSTAR tool, a validated 11-item tool to assess the quality of systematic reviews[17]. The tool will also be applied to non-systematic reviews in order to assess the quality of included reviews against the ‘gold standard’ of a systematic review. Non-applicable items will not be weighted in order to avoid undue judgements on the quality of non-systematic reviews raised by the (potential) non-applicability of AMSTAR items.

There is currently no consensus among qualitative researchers on the role of quality criteria and how they should be applied, and there is ongoing debate about how study quality should be assessed for the purposes of systematic reviews[13, 18]. Prior to the review process, a selective literature review has been conducted by the review team to identify the most suitable assessment tool for the quality appraisal of qualitative studies, which will be included in our systematic review. We followed the Cochrane guidance for “Critical Appraisal of Qualitative Research”[19, 20] and agreed on the use of the *Critical Appraisal Skills Programme (CASP)* for qualitative studies (see Additional file8 for CASP Screening Questions). The reasons for applying CASP are as follows:

- It is widely used in similar reviews and recommended by Cochrane guidance and the guidance of the Center for Reviews and Dissemination (CRD).
- It contains only 10 items for rapid evaluation.
- It is suitable for different types of qualitative studies.

The critical appraisal process will not lead to exclusion of papers, but rather serves as one of several other criteria to evaluate the body of evidence.

### Analysis of findings

#### Summary table

One or more tables will be drawn up containing condensed information from the data extraction forms (i.e., a description of included studies, study populations, methods, analyzed health outcomes/health-care domains, results, and quality).

#### Evidence map

In the next step, we will create a thematic and conceptual evidence map to illustrate the research landscape and identify research gaps. To this end, all included articles will be grouped and categorized by year of data collection (pre- vs. post-1993), research design (quantitative/qualitative/mixed methods), and the analyzed health conditions and/or health-care domains. In its descriptive way, this map will address objectives 1 and 3 by providing a systematic description of available research. The body of evidence (in particular, related to objective 3) will be evaluated by the following criteria: the number of studies analyzing the same outcome(s) (quantitative studies) or exploring the same topic (qualitative studies), overall quality and risk of bias (quantitative studies)/credibility (qualitative studies), external validity (quantitative studies)/saturation and transferability (qualitative studies), and consistency of findings across studies. Any modifications to this protocol will be made transparent and documented in the final review report.

#### Evidence synthesis

As our review question dictates the inclusion of many different research designs, we will follow the recommendation to use a primarily “narrative synthesis”[21] to answer review question 2. Despite the absence of procedures and standards, the narrative synthesis allows flexibility and coping with large evidence base, comprising diverse evidence types. Beginning with a quantitative analysis, the included studies will be organized and presented in logical categories by study design. We decided to apply a multi-level approach (according to Cochrane Handbook for Systematic Reviews of Interventions[20]), where quantitative evidence (synthesis 1) and qualitative evidence (synthesis 2) are synthesized as separate streams and the product of each synthesis is then combined (synthesis 3).

If possible, we will conduct a meta-analysis to summarize the findings of quantitative studies which analyze the same outcome.

As there are no standard approaches for the synthesis of qualitative data[13], we will combine a descriptive synthesis with a narrative elaboration of the patterns identified in qualitative studies. Narrative descriptions of each included article will provide a short, clear summary of information on a range of process and outcome measures. We will then combine separate elements to form a coherent whole, a synthesized finding of qualitative research (interpretative synthesis).

In the third synthesis, we will integrate the findings of all primary studies (quantitative and qualitative ones), taking into account variations in study design, context, and study quality.

This final configuration of synthesized findings will be a summary of knowledge about the health status of and health-care provision to asylum seekers in Germany, as generated by empirical studies.

**Figure 1:**
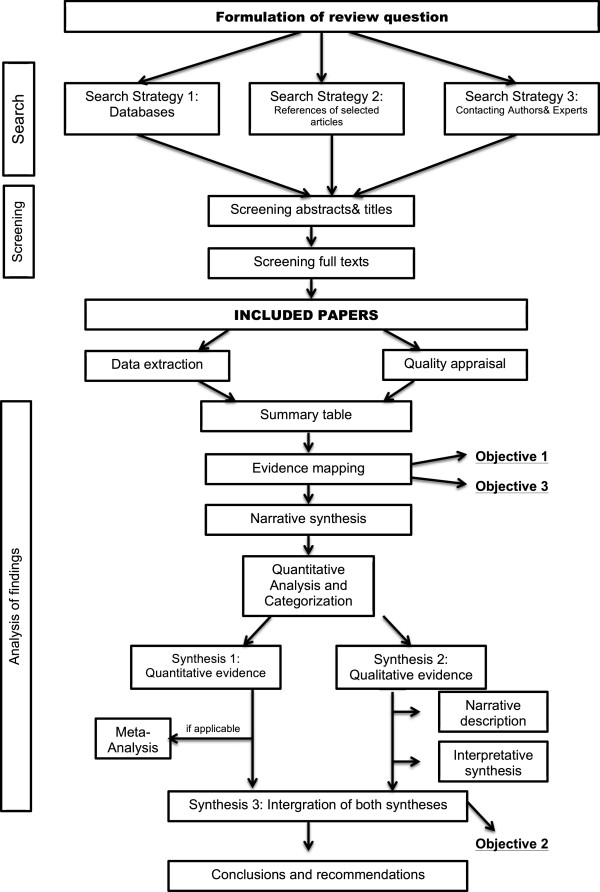
**Design and process of the systematic review on health status of and health-care provision to asylum seekers in Germany.**

The overall design and process of this review is shown in Figure 1.

## Discussion

This systematic review will provide the first evidence map and synthesis of available research findings on the health status of and health-care provision to asylum seekers in Germany. Such an overview is paramount to avoid redundant research, identify knowledge gaps, and consolidate existing knowledge. We hope that the results will help guide future research on this topic and help shape a future research agenda to improve the health and health care of this marginalized population. The results of our review could also be used to identify potential targets and areas for interventions aimed at treating or preventing certain conditions, or mitigating the effects of individual or structural risk factors on asylum seekers’ health.

The strength of this anticipated review lies in a clear protocol with eligibility criteria, a transparent and systematic search strategy, and advanced approaches for screening, extracting, and appraising the available research. The review follows clear steps for the analysis of findings by evidence mapping and narrative description of results. We include both German and English quantitative and qualitative studies published both before and after 1993, when asylum policy changes were made. Thus, we ensure to cover a wide range of relevant information.

As such, this review will provide a detailed and reliable overview of the field for future research and will identify evidence gaps which require further exploration. We further anticipate that information gained by the review will help health professionals and policy makers to better understand needs in health-care provision which need to be addressed.

To ensure that our results will be accessible to policy makers, health-care providers, and researchers and promote further discussion, we will publish this review in an open access journal and disseminate the findings via conferences, civil society organizations, and academic institutions.

## Electronic supplementary material

Additional file 1:**Search strategy for databases.** This shows the searched databases with the according search term. (PDF 39 KB)

Additional file 2:**Sensitivity and specificity.** Lists of all references which serve as a test set to assess sensitivity and specificity of the prior conducted screening. (PDF 72 KB)

Additional file 3:**Data extraction form-quantitative studies.** The data extraction form which will be used to analyze the included quantitative studies. (XLS 71 KB)

Additional file 4:**Data extraction form-qualitative studies.** The data extraction form which will be used to analyze the included qualitative studies. (XLS 72 KB)

Additional file 5:**Data extraction form-reviews.** The data extraction form which will be used to analyze the included reviews. (XLS 72 KB)

Additional file 6:**EPHPP Quality Assessment Tool for Quantitative Studies.** The tool to assess the quality of the quantitative studies. (PDF 75 KB)

Additional file 7:**McGill Mixed-Methods Appraisal Tool for Mixed-Methods Studies.** The tool to assess the quality of the mixed-methods studies. (PDF 220 KB)

Additional file 8:**Critical Appraisal Skills Programme (CASP).** A tool to assess the quality of the qualitative studies. (PDF 230 KB)

## Abbreviations

IBSS: International Bibliography of Social Sciences
IDP: internally displaced people
ASSIA: Applied Social Sciences Index and Abstracts
CINAHL: Cumulative Index to Nursing and Allied Health Literature
DNB: German National Library
UNHCR: United Nations High Commissioner for Refugees
WHO: World Health Organization
EPPI: The Evidence for Policy and Practice Information and Co-ordinating Centre
CRD: Centre for Reviews and Dissemination.
