# Supplementary material for: Health status of and health-care provision to asylum seekers in Germany: protocol for a systematic review and evidence mapping of empirical studies
Source: Syst Rev. 2014 Nov 29;3:139. doi: 10.1186/2046-4053-3-139 (PMC4259011; doi:10.1186/2046-4053-3-139)
Supplement: Supplementary file 1 — Additional file 1:Search strategy for databases. This shows the searched databases with the according search term. (PDF 39 KB) [file 13643_2014_305_MOESM1_ESM.pdf]

# Additional File 1: Search Strategy

Supplement to: Schneider C, Mohsenpour A, Joos S, Bozorgmehr K: Health status of and healthcare provision to asylum seekers in Germany: Protocol for a systematic review and evidence mapping of empirical studies

## Databases searched on DD/MM/YYYY by AM and CS

| **Database** | **Search Term** | Link |
|---|---|---|
| Pubmed | ((refugee* OR asylum*) AND (health* OR access OR utilization) AND german*) | http://www.ncbi.nlm.nih.gov/pubmed/ |
| ISI Web of Science | ((refugee* OR asylum*) AND (health* OR access OR utilization) AND german*) | https://webofknowledge.com |
| IBSS | ((refugee* OR asylum*) AND (health* OR access OR utilization) AND german*) | http://search.proquest.com/ibss |
| Medpilot | (refugee* OR asylum*) AND (health* OR access OR utilization) AND german* | http://www.medpilot.de/ |
| Medpilot | (Asyl* OR Flüchtling*) AND gesundheit* | http://www.medpilot.de/ |
| Sociological Abstracts | (refugee* OR asylum*) AND (health* OR access OR utilization) AND german* | http://search.proquest.com/socabs |
| Social Science Citation Index | (refugee* OR asylum*) AND (health* OR access OR utilization) AND german* | https://webofknowledge.com |
| CINAHL | (refugee* OR asylum*) AND (health* OR access OR utilization) AND german* | via EBSCO Host |
| ASSIA | (refugee* OR asylum*) AND (health* OR access OR utilization) AND german* | http://search.proquest.com/assia |
| Worldwide Political Science Abstracts | (refugee* OR asylum*) AND (health* OR access OR utilization) AND german* | http://search.proquest.com/wpsa |
| Sowiport | Flüchtling* OR asyl* AND gesundheit* | http://www.gesis.org/sowiport/ |
| Sowiport | refugee* OR asylum* AND health* AND german* | http://www.gesis.org/sowiport/ |
| Deutsche National-bibliothek | Flüchtling* OR asyl* AND gesundheit* | http://dnb.dnb.de/ |
| Google | (Asyl* ODER Flüchtling*) UND (Gesundheit ODER Gesundheitsversorgung) UND (Studie ODER qual* ODER quant*) | www.google.com |
| Google | (refugee* OR asylum*) AND (health* OR access OR utilization OR use*) AND german* | www.google.com |
| Google | (refugee* OR asylum*) AND health* AND study* AND german* | www.google.com |
