# Supplementary material for: Health status of and health-care provision to asylum seekers in Germany: protocol for a systematic review and evidence mapping of empirical studies
Source: Syst Rev. 2014 Nov 29;3:139. doi: 10.1186/2046-4053-3-139 (PMC4259011; doi:10.1186/2046-4053-3-139)
Supplement: Supplementary file 2 — Additional file 2:Sensitivity and specificity. Lists of all references which serve as a test set to assess sensitivity and specificity of the prior conducted screening. (PDF 72 KB) [file 13643_2014_305_MOESM2_ESM.pdf]

## Additional File 2: Test Set- References

Supplement to: Schneider C, Mohsenpour A, Joos S, Bozorgmehr K: Health status of and healthcare provision to asylum seekers in Germany: Protocol for a systematic review and evidence mapping of empirical studies

### Test Set – References


1. Pross C: **Third Class Medicine: Health Care for Refugees in Germany.** *Health and Human Rights* 1998, **3:**40-53.
2. Behrensen B, Groß V: **Auf dem Weg in ein "normales Leben"? Eine Analyse der gesundheitlichen Situation von Asylsuchenden in der Region Osnabrück.** In *Auf dem Weg in ein "normales Leben"? Eine Analyse der gesundheitlichen Situation von Asylsuchenden in der Region Osnabrück*. Osnabrück: 2004.
3. Norredam M, Mygind A, Krasnik A: **Access to health care for asylum seekers in the European Union--a comparative study of country policies.** *Eur J Public Health* 2006, **16:**286-290.
4. Aumüller J, Bretl C: **Die kommunale Integration von Flüchtlingen in Deutschland.** In *Die kommunale Integration von Flüchtlingen in Deutschland*. 2008.
5. Razum O, Zeeb H, Meesmann U, Schenk L, Bredehorst M, Brzoska P, Dercks T, Glodny S, Menkhaus B, Salman R, et al: **Schwerpunktbericht der Gesundheitsberichterstattung des Bundes.** In *Schwerpunktbericht der Gesundheitsberichterstattung des Bundes*. Berlin: Robert Koch- Institut; 2008.
6. Riesberg A, Wörz M: **Quality in and Equality of Access to healthcare- services in EU - Country Report for Germany.** In *Quality in and Equality of Access to healthcare- services in EU - Country Report for Germany* eds. City: European Commission; 2008.
7. von Lersner U, Wiens U, Elbert T, Neuner F: **Mental health of returnees: refugees in Germany prior to their state-sponsored repatriation.** *BMC international health and human rights* 2008, **8:**8.
8. Jung F: **Das Bremer Modell- Gesundheitsversorgung Asylsuchender.** Gesundheitsamt Bremen; Bremen: 2011.
9. Dudareva S, Barth A, Paeth K, Krenz-Weinreich A, Layer F, Delere Y, Eckmanns T: **Cases of community-acquired meticillin-resistant Staphylococcus aureus in an asylum seekers centre in Germany, November 2010.** *Euro Surveill* 2011, **16**.
10. Chauvin P, Simonnot N: **Access to health care for vulnerable groups in the European Union in 2012. An overview of the condition of persons excluded from healthcare systems in the EU.** Médecins du monde; n.p.g.: 2012.
11. Takla A, Barth A, Siedler A, Stocker P, Wichmann O, Delere Y: **Measles outbreak in an asylum-seekers' shelter in Germany: comparison of the implemented with a hypothetical containment strategy.** *Epidemiology and infection* 2012, **140:**1589-1598.
