# Supplementary material for: Health status of and health-care provision to asylum seekers in Germany: protocol for a systematic review and evidence mapping of empirical studies
Source: Syst Rev. 2014 Nov 29;3:139. doi: 10.1186/2046-4053-3-139 (PMC4259011; doi:10.1186/2046-4053-3-139)
Supplement: Supplementary file 7 — Additional file 7:McGill Mixed-Methods Appraisal Tool for Mixed-Methods Studies. The tool to assess the quality of the mixed-methods studies. (PDF 220 KB) [file 13643_2014_305_MOESM7_ESM.pdf]

Types of mixed methods study components or primary studies	Methodological quality criteria (see tutorial for definitions and examples)	Responses			
		Yes	No	Can't tell	Comments
**Screening questions (for all types)**	• Are there clear qualitative and quantitative research questions (or objectives*), or a clear mixed methods question (or objective*)?				
	• Do the collected data allow address the research question (objective)? E.g., consider whether the follow-up period is long enough for the outcome to occur (for longitudinal studies or study components).				
	Further appraisal may be not feasible or appropriate when the answer is 'No' or 'Can't tell' to one or both screening questions.				
**1. Qualitative**	1.1. Are the sources of qualitative data (archives, documents, informants, observations) relevant to address the research question (objective)?				
	1.2. Is the process for analyzing qualitative data relevant to address the research question (objective)?				
	1.3. Is appropriate consideration given to how findings relate to the context, e.g., the setting, in which the data were collected?				
	1.4. Is appropriate consideration given to how findings relate to researchers' influence, e.g., through their interactions with participants?				
**2. Quantitative randomized controlled (trials)**	2.1. Is there a clear description of the randomization (or an appropriate sequence generation)?				
	2.2. Is there a clear description of the allocation concealment (or blinding when applicable)?				
	2.3. Are there complete outcome data (80% or above)?				
	2.4. Is there low withdrawal/drop-out (below 20%)?				
**3. Quantitative non-randomized**	3.1. Are participants (organizations) recruited in a way that minimizes selection bias?				
	3.2. Are measurements appropriate (clear origin, or validity known, or standard instrument; and absence of contamination between groups when appropriate) regarding the exposure/intervention and outcomes?				
	3.3. In the groups being compared (exposed vs. non-exposed; with intervention vs. without; cases vs. controls), are the participants comparable, or do researchers take into account (control for) the difference between these groups?				
	3.4. Are there complete outcome data (80% or above), and, when applicable, an acceptable response rate (60% or above), or an acceptable follow-up rate for cohort studies (depending on the duration of follow-up)?				
**4. Quantitative descriptive**	4.1. Is the sampling strategy relevant to address the quantitative research question (quantitative aspect of the mixed methods question)?				
	4.2. Is the sample representative of the population understudy?				
	4.3. Are measurements appropriate (clear origin, or validity known, or standard instrument)?				
	4.4. Is there an acceptable response rate (60% or above)?				
**5. Mixed methods**	5.1. Is the mixed methods research design relevant to address the qualitative and quantitative research questions (or objectives), or the qualitative and quantitative aspects of the mixed methods question (or objective)?				
	5.2. Is the integration of qualitative and quantitative data (or results*) relevant to address the research question (objective)?				
	5.3. Is appropriate consideration given to the limitations associated with this integration, e.g., the divergence of qualitative and quantitative data (or results*) in a triangulation design?				
	Criteria for the qualitative component (1.1 to 1.4), and appropriate criteria for the quantitative component (2.1 to 2.4, or 3.1 to 3.4, or 4.1 to 4.4), must be also applied.				
